# Author Correction: Orbital-selective effect of spin reorientation on the Dirac fermions in a non-charge-ordered kagome ferromagnet Fe_3_Ge

**DOI:** 10.1038/s41467-024-55127-8

**Published:** 2024-12-09

**Authors:** Rui Lou, Liqin Zhou, Wenhua Song, Alexander Fedorov, Zhijun Tu, Bei Jiang, Qi Wang, Man Li, Zhonghao Liu, Xuezhi Chen, Oliver Rader, Bernd Büchner, Yujie Sun, Hongming Weng, Hechang Lei, Shancai Wang

**Affiliations:** 1 Leibniz Institute for Solid State and Materials Research, IFW Dresden, 01069 Dresden, Sachsen Germany; 2 Helmholtz-Zentrum Berlin für Materialien und Energie, Albert-Einstein-Straße 15, 12489 Berlin, Germany; 3 Joint Laboratory “Functional Quantum Materials” at BESSY II, 12489 Berlin, Germany; 4 Beijing National Laboratory for Condensed Matter Physics and Institute of Physics, Chinese Academy of Sciences, Beijing, 100190 China; 5 University of Chinese Academy of Sciences, Beijing, 100049 China; 6 Department of Physics, Key Laboratory of Quantum State Construction and Manipulation (Ministry of Education), and Beijing Key Laboratory of Opto-electronic Functional Materials & Micro-nano Devices, Renmin University of China, Beijing, 100872 China; 7 School of Physical Science and Technology, ShanghaiTech University, Shanghai, 201210 China; 8 ShanghaiTech Laboratory for Topological Physics, ShanghaiTech University, Shanghai, 201210 China; 9 School of Information Network Security, People’s Public Security University of China, Beijing, 100038 China; 10 Institute of High-Pressure Physics and School of Physical Science and Technology, Ningbo University, Ningbo, 315211 China; 11 Shanghai Institute of Applied Physics, Chinese Academy of Sciences, Shanghai, 201800 China; 12 Institute of Solid State and Materials Physics, TU Dresden, 01062 Dresden, Sachsen Germany; 13 Department of Physics and Guangdong Basic Research Center of Excellence for Quantum Science, Southern University of Science and Technology (SUSTech), Shenzhen, 518055 China; 14 Quantum Science Center of Guangdong-Hong Kong-Macao Greater Bay Area (Guangdong), Shenzhen, 518045 China; 15 Institute of Advanced Science Facilities, Shenzhen, Guangdong 518107 China; 16 Songshan Lake Materials Laboratory, Dongguan, Guangdong 523808 China

**Keywords:** Electronic properties and materials, Topological matter, Magnetic properties and materials

Correction to: *Nature Communications* 10.1038/s41467-024-53343-w, published online 13 November 2024

In the Acknowledgements section, the grant number for National Natural Science Foundation of China ‘12174443’ should have read ‘12274459’. Second, the grant number for the Ministry of Science and Technology of China ‘2018YFE0202600’ should have read ‘2023YFA1406500’. The original article has been corrected.

