# Supplementary material for: Orbital-selective effect of spin reorientation on the Dirac fermions in a non-charge-ordered kagome ferromagnet Fe3Ge
Source: Nat Commun. 2024 Nov 13;15:9823. doi: 10.1038/s41467-024-53343-w (PMC11561085; doi:10.1038/s41467-024-53343-w)
Supplement: Supplementary file 1 — Supplementary Information [file 41467_2024_53343_MOESM1_ESM.pdf]

Supplementary Information

Orbital-selective effect of spin reorientation on the Dirac fermions in a non-charge-ordered kagome ferromagnet Fe_3Ge

Lou *et al.*

Contents

- **Figure S1: Low-energy electron diffraction patterns**
- **Figure S2: X-ray photoelectron spectroscopy measurements**
- **Figure S3: Comparison between the DFT calculations in the paramagnetic and uniaxial ferromagnetic states**
- **Figure S4: Photon-energy-dependent dispersions**
- **Figure S5: DFT calculated Fermi surfaces**
- **Figure S6: Two energy bands passing through the DP1 along the *K*-*M* direction**
- **Figure S7: Two band dispersions forming the DP1 along the Γ-*K* direction**
- **Figure S8: Observation of the flat band**
- **Figure S9: Spin-orbit-coupling-induced gap at the DP1**
- **Figure S10: Orbital-resolved Fe-3*d* band structure**
- **Figure S11: Orbital-projected DFT calculations**
- **Figure S12: Density-of-states calculations**
- **Figure S13: DFT calculations without the spin-orbit coupling effect**
- **Figure S14: Correspondence between the ARPES spectra and the renormalized DFT calculations**

- **Table S1-S3: Representation analysis of different magnetic configurations**
- **Table S4: Quantitative comparison between ARPES and DFT**
- **Note 1: Stable stoichiometry and detailed analysis of the X-ray photoelectron spectroscopy data**
- **Note 2: Different band degeneracies between different magnetic states**
- **Note 3: Observation of the two branches of DP1 along the Γ-*K* direction in the second Brillouin zone**
- **Note 4: Reproducibility, temperature evolution, and quantitative analysis of the DP1 gap**
- **Note 5: Negligible contribution of spin-orbit coupling to the Dirac gap at DP2**
- **Note 6: Examining whether the vHSs can induce electronic instabilities**

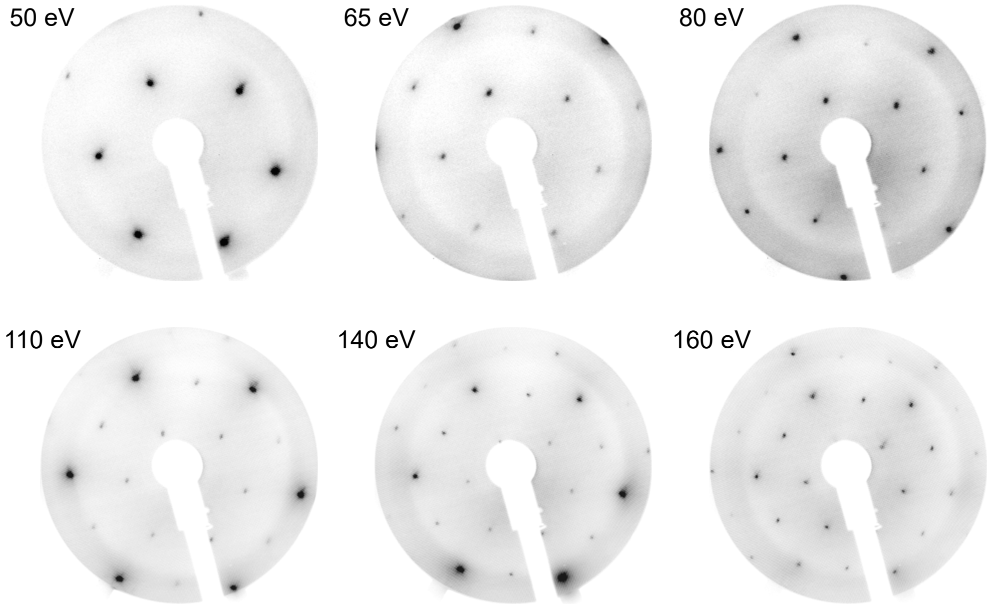

Figure S1 | Low-energy electron diffraction (LEED) patterns. LEED images of the treated Fe_3Ge-(001) surface obtained by the electron energies of 50, 65, 80, 110, 140, and 160 eV at room temperature, respectively. There are no additional superstructure spots, elucidating the absence of surface reconstruction.

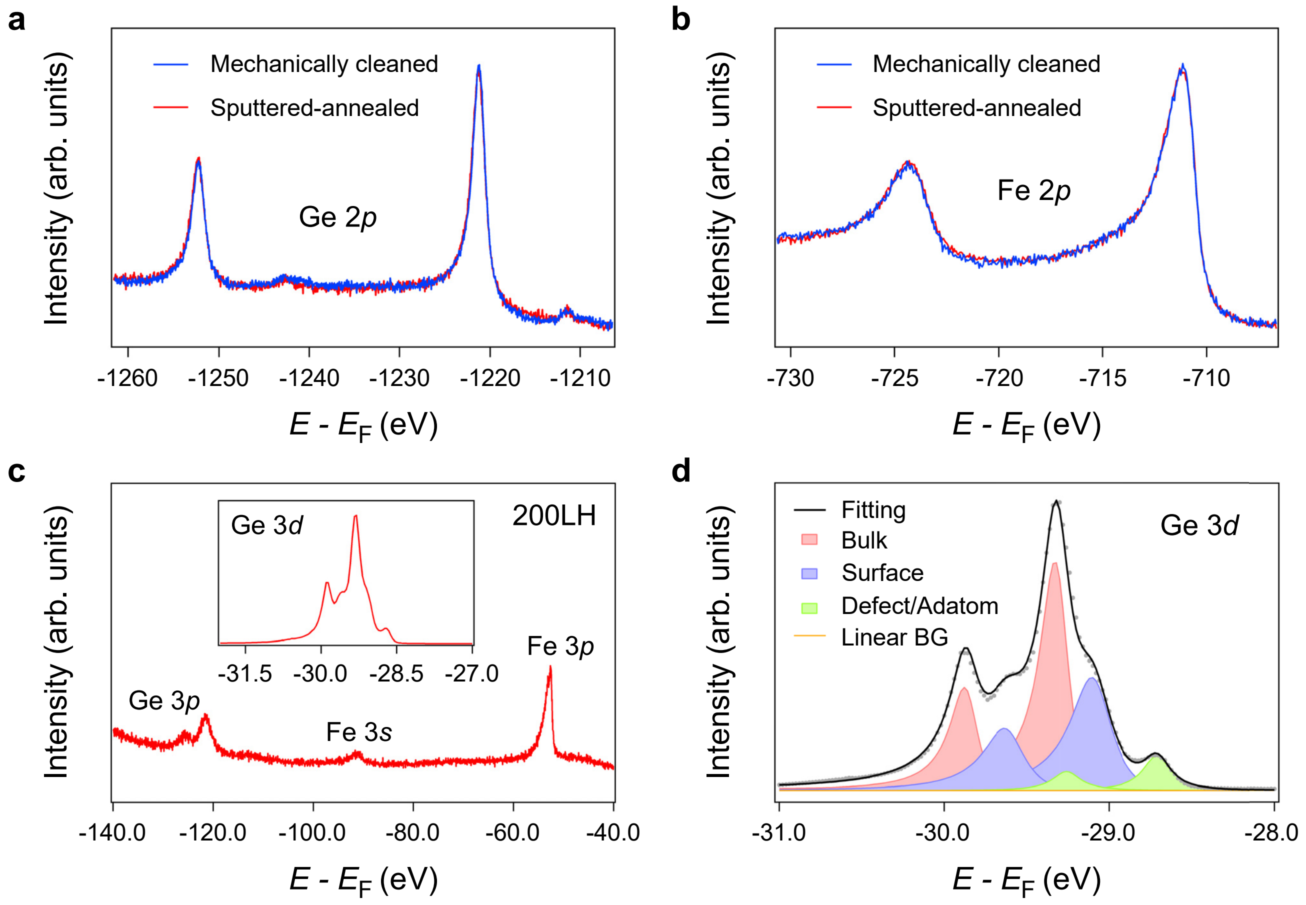

Figure S2 | X-ray photoelectron spectroscopy (XPS) measurements. a,b, XPS spectra of the Ge 2*p* (**a**) and Fe 2*p* (**b**) core levels measured on the mechanically cleaned and sputtered-annealed crystal surfaces of Fe_3Ge using an Al K_α X-ray source, respectively. **c,** Core-level photoemission spectra of Fe_3Ge recorded at $h\nu$ = 200 eV (linear horizontal (LH) polarization). The core-level spectra of the Ge 3*p*, Fe 3*s*, Fe 3*p*, and Ge 3*d* (inset) have been identified. **d,** Multipeak fitting of the Ge 3*d* core-level spectra in **c** by six Lorentzian peaks (shades) with a linear background (orange line). The asymmetry was introduced into the Lorentzian peaks to capture the tail on the high binding energy side.

Supplementary Note 1: Stable stoichiometry and detailed analysis of the XPS data

To demonstrate the negligible effect of sputtering and annealing procedures on the sample stoichiometry, we conducted the comparative XPS measurements on the surfaces without and with the sputter-anneal cycles. First, we mechanically cleaned *in situ* the surface of an as-grown Fe_3Ge single crystal by means of a diamond needle file, and recorded the XPS spectra on it; then, we processed this clean surface using the same sputter-anneal cycles as in the main text, and performed similar XPS measurements. As shown in Fig. S2a,b, the overall line shapes from these two surfaces are quite similar for both the Ge-2*p* and Fe-2*p* core levels, implying a negligible change of the sample composition. Moreover, we do not observe any additional reconstructions in the LEED patterns (Fig. S1) and the ARPES spectra show a good overall agreement with the DFT calculations. Therefore, we believe that the stoichiometry of Fe_3Ge crystals is little affected by the sputter-anneal treatments.

On the other hand, to understand the components involved in the Ge-3*d* core level (inset of Fig. S2c), we carried out the quantitative fit of the XPS spectra. As shown in Fig. S2d, the spectra can be well reproduced by three spin-orbit split doublets with a linear background. The values of spin-orbit splitting (~0.55 eV) and branching ratio (~1.81) of each doublet are comparable to the typical values of Ge 3*d* states reported in other materials, like the Ge(111) [1]. In general, the XPS intensity ratio between bulk and surface components depends sensitively on the emission angle (the angle between the sample surface normal and the lens axis of analyzer); as the emission angle decreases, the bulk sensitivity of the measurements is enhanced, leading to the enhancement of the bulk-to-surface intensity ratio. In the present case, our XPS measurements were performed near the normal emission angle (0°), the observed difference in the intensity of bulk and surface components is therefore reasonable. Meanwhile, according to our fitting results, the energy difference (absolute value) between bulk and surface components is about 0.24 eV, also agreeing well with the reported values in Ge(111) [1]. Regarding the doublet with tiny intensities at lower binding energies (green shades in Fig. S2d), we infer that it comes from (i) the defects during bulk crystal growth and/or (ii) the defects/adatoms on the surface introduced by the polishing and/or the sputter-anneal treatments. Nonetheless, it is noted that such defects made no noticeable contribution to our ARPES spectra.

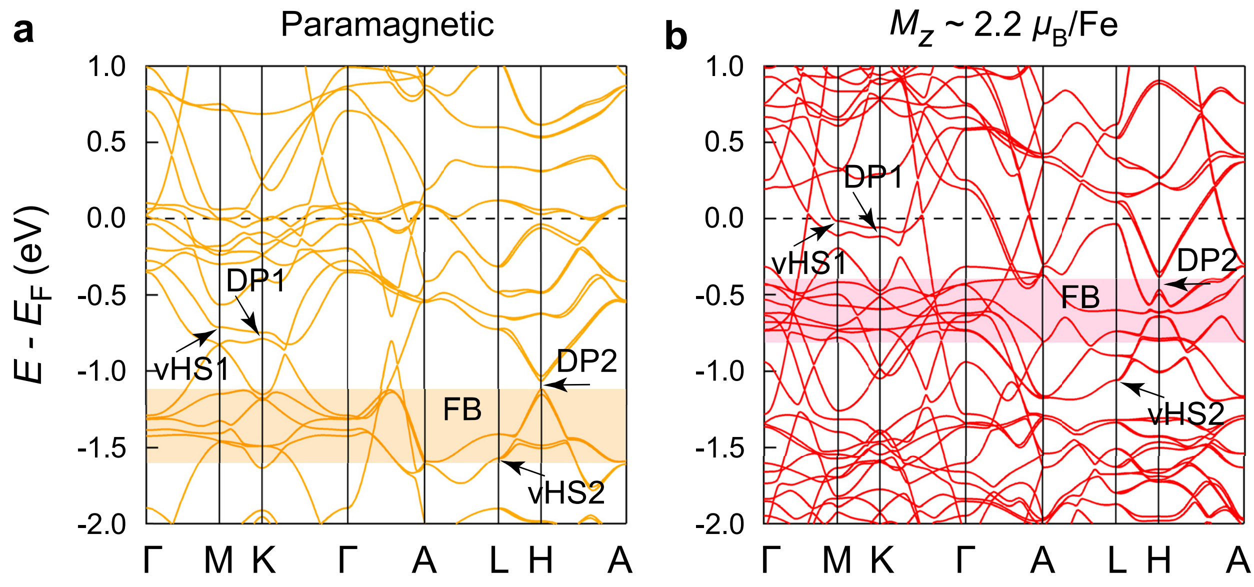

Figure S3 | Comparison between the DFT calculations in the paramagnetic (PM) and uniaxial ferromagnetic (FM) states. a, DFT calculated band structure for the PM phase of Fe_3Ge. **b,** Same as **a** for the FM Fe_3Ge with the Fe moments ($\mu_{Fe} \approx 2.2\ \mu_B$) aligned along the *z* axis. The Dirac point 1,2 (DP1,2), van Hove singularity 1,2 (vHS1,2), and flat band (FB) regions are marked out.

Supplementary Note 2: Different band degeneracies between different magnetic states

As shown in Figs. 1e-g and S3, along the Γ-*A*, *L*-*H*, and *H*-*A* lines, the small degeneracy lifting of some bands appears in the PM state and FM state with the Fe moments aligned along the *z* axis, while this does not appear when the FM moments lie along the *x* and *y* axes. The magnetic configurations of Fe_3Ge with different directions lead to different magnetic space groups (MSGs), making the little groups on certain high-symmetry paths with different symmetries. As shown in Tables S1-S3, since the bands of Fe_3Ge with the FM moments along *x* ([100]), *y* ([010]) and *z* ([001]) directions or in the PM phase have different little groups along the Γ-*A*, *L*-*H*, and *H*-*A* paths, they have different degeneracies on different paths.

Γ-*A* (0, 0, w)

Phase	MSG	Unitary subgroup	Magnetic little co-group	Little co-group	Little group
PM	$P6_3$/mmc1' (194.264)	194	6/m'mm	6mm	C6v
FM[100]	Cmc'm' (63.463)	12	m'm2'	m	Cs
FM[010]	Cm'cm' (63.464)	15	m'm2'	m	Cs
FM[001]	$P6_3$/mm'c' (194.270)	176	62'2'	6	C6

L-*H* (u, v, 1/2)

Phase	MSG	Unitary subgroup	Magnetic little co-group	Little co-group	Little group
PM	$P6_3$/mmc1' (194.264)	194	2'/m	m	C2v
FM[100]	Cmc'm' (63.463)	12	2'	1	C1
FM[010]	Cm'cm' (63.464)	15	2'	1	C1
FM[001]	$P6_3$/mm'c' (194.270)	176	m	m	Cs

H-*A* (u, u, 1/2)

Phase	MSG	Unitary subgroup	Magnetic little co-group	Little co-group	Little group
PM	$P6_3$/mmc1' (194.264)	194	m'mm	mm2	C2v
FM[100]	Cmc'm' (63.463)	12	/	/	C1
FM[010]	Cm'cm' (63.464)	15	/	/	C1
FM[001]	$P6_3$/mm'c' (194.270)	176	m'm2'	m	Cs

Table S1-S3 | Representation analysis of different magnetic configurations. MSGs, unitary subgroups, magnetic little co-groups, little co-groups, and little groups of different magnetic configurations along the Γ-*A* (**Table S1**), *L*-*H* (**Table S2**) and *H*-*A* (**Table S3**) paths, respectively.

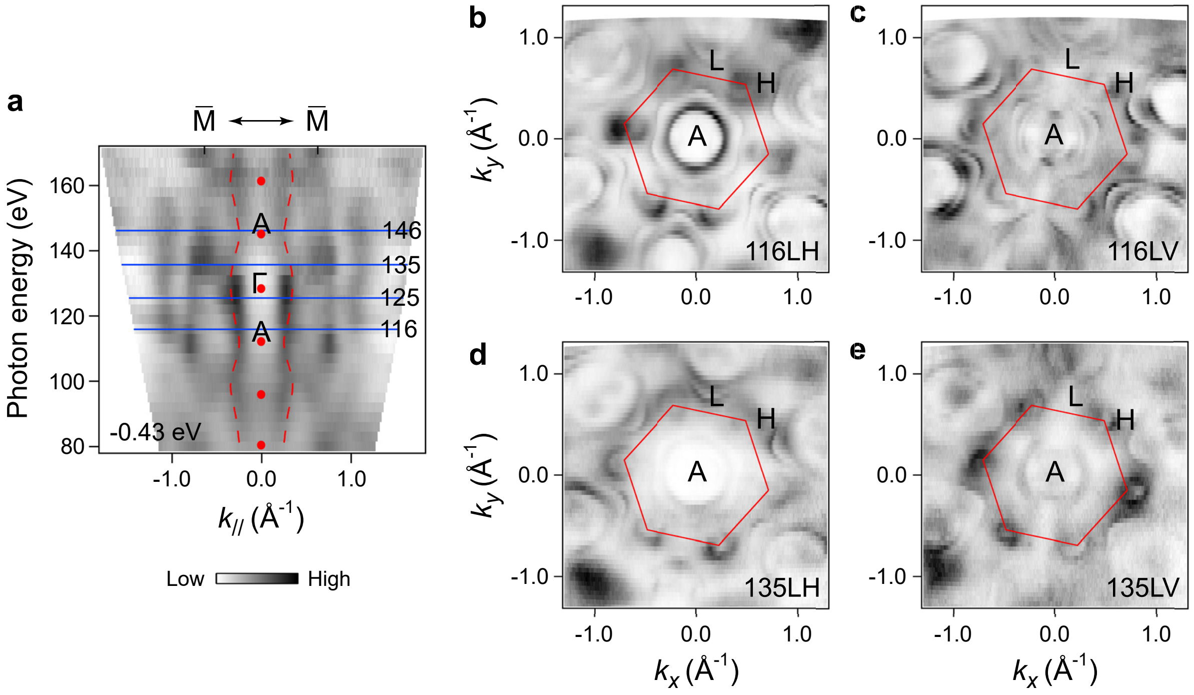

Figure S4 | Photon-energy-dependent dispersions. a, ARPES intensity plot in the hv-$k_{//}$ plane at the energy of −0.43 eV, with $k_{//}$ oriented along the $\bar{\Gamma}$-$\bar{M}$ direction. The red dashed curves are guides to the eye for the intensity modulation along k_z. **b,c,** Fermi surface (FS) mappings taken by the 116-eV photons (close to $k_z = \pi$ plane) with LH (**b**) and linear vertical (LV) (**c**) polarizations, respectively. **d,e,** Same as **b,c** measured by the 135-eV photons. Similar to the FS recorded at hv = 146 eV (Fig. 2b in the main text), one can clearly see the Dirac pocket around H point in **b-e**.

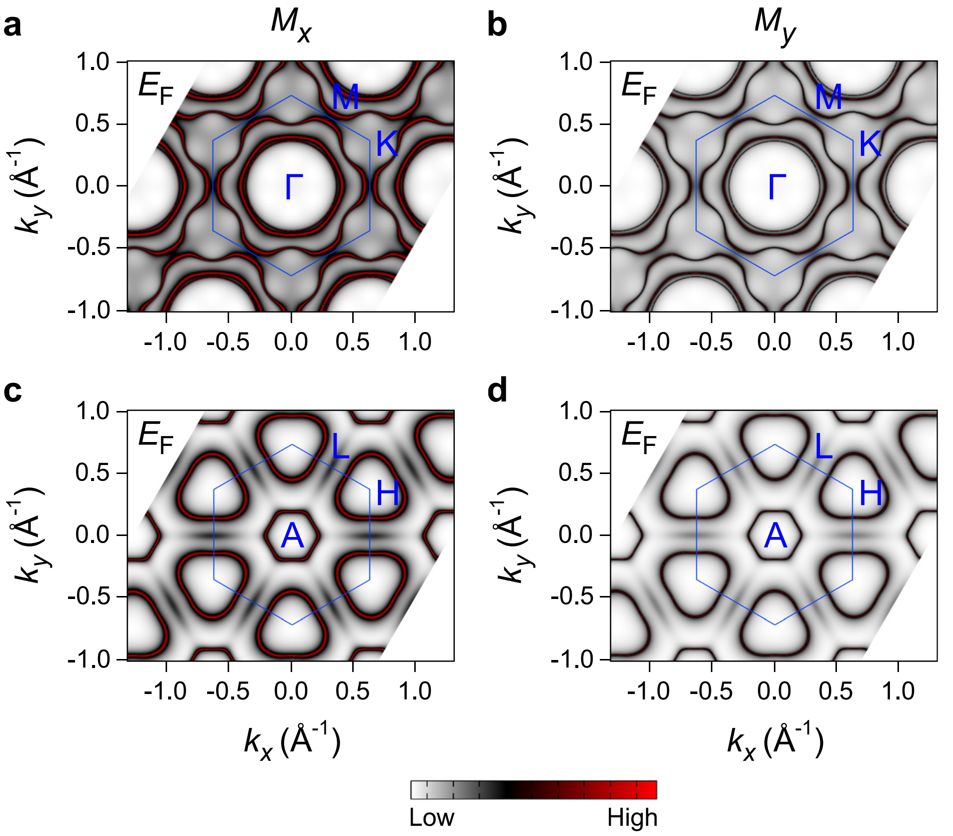

Figure S5 | DFT calculated FSs. a, DFT calculated bulk FSs of Fe_3Ge in the k_z = 0 plane. The calculations were carried out by considering a FM moment ($\mu_{Fe} \approx 2.2\mu_B$) aligned along the *x* axis. **b,** Same as **a** with the FM moment along the *y* axis. **c,d,** Same as **a,b** in the $k_z = \pi$ plane.

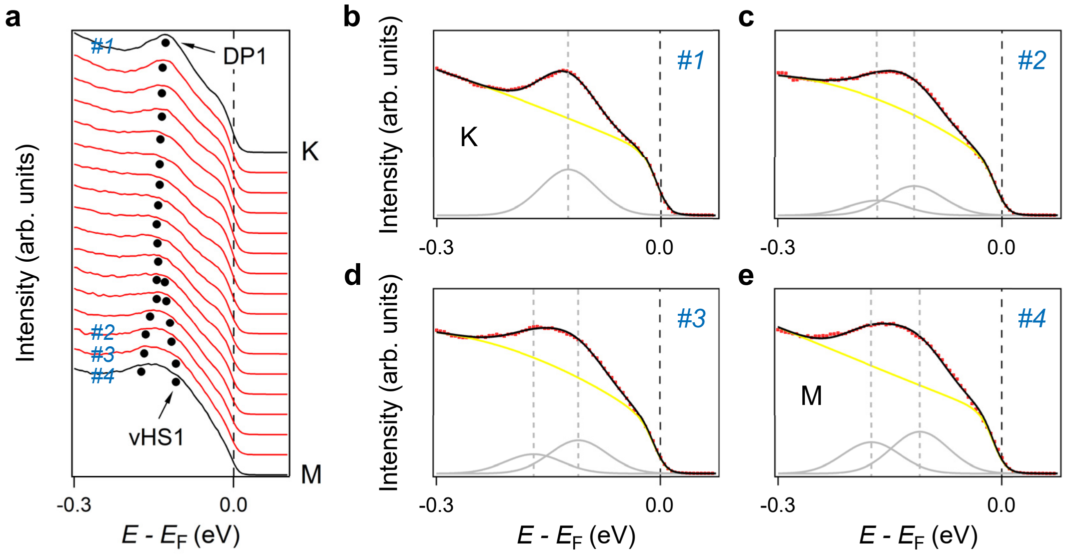

Figure S6 | Two energy bands passing through the DP1 along the *K*-*M* direction. a, Energy distribution curves (EDCs) of the ARPES spectra taken along the *K*-*M* direction. The black solid circles indicate the band dispersions associated with the DP1. The separation between these two bands can hardly be resolved in the momentum range from ~1/3*MK* to *K* point. **b-e,** Quantitative fitting of the individual EDCs (#1-#4 in **a**) by using one Gaussian peak (#1, **b**) and two Gaussian peaks (#2-#4, **c-e**), respectively. The Gaussian peaks are displayed as the grey solid curves. The background (yellow solid curves) is modeled by considering a polynomial function together with the Fermi-Dirac distribution. The fitting results are superimposed as the black solid curves. To ensure the validity of using two peaks in the fits of the EDCs close to *M* point, the full width at half maximum (FWHM) of each Gaussian peak in **c-e** is constrained to the same value (~0.09 eV) as that in **b**; as a result, the obtained good fits can validate our identification of the double-hump spectral feature near *M* point.

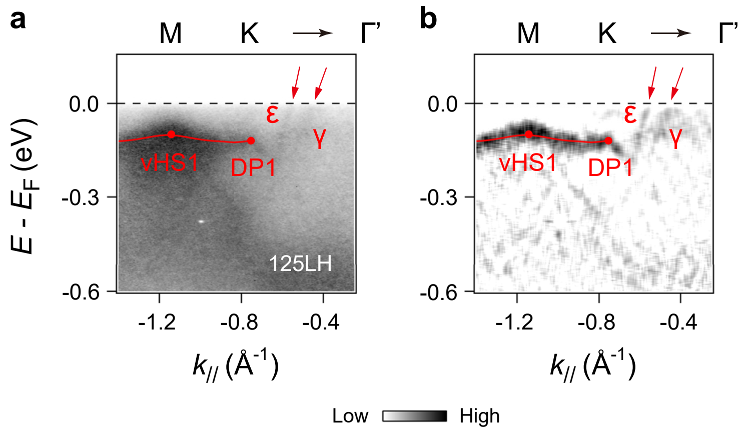

Figure S7 | Two band dispersions forming the DP1 along the Γ-*K* direction. a,b, ARPES intensity plot (**a**) and corresponding second derivative plot (**b**) recorded along the Γ’-*K*-*M* direction with the 125-eV photons (LH polarization), respectively. Γ’ denotes the center of the second Brillouin zone (BZ). The red arrows indicate the two bands (*γ* and *ε*) forming the DP1 along the Γ’-*K* direction. The red curves are guides to the eye for the vHS1 band along the *K*-*M*-*K* direction and its connecting to the DP1.

Supplementary Note 3: Observation of the two branches of DP1 along the Γ-*K* direction in the second BZ

Regarding the two branches of DP1 along the Γ-*K* direction, one is experimentally revealed (*γ* band, Fig. 3a), the other (denoted as *ε* band) is not visible in the first BZ but in the second BZ due to matrix element effects. This is justified by the FS topologies in experiments (Fig. 2a). More specifically, according to the DFT calculated FSs (Fig. 2c) and band structures (Fig. 3b) in the k_z = 0 plane, one obtains that the two branches of DP1 along the Γ-*K* direction contribute to the two hole-like Fermi pockets around Γ point. These two Fermi pockets are clearly observed in our experiments (Fig. 2a); moreover, the outer pocket near the Γ-*K* direction, which corresponds to the *ε* band, is vanishingly weak in the first BZ while is more visible in the second BZ due to matrix element effects. To validate the presence of *ε* band along the Γ-*K* direction, we measured the ARPES spectra in the second BZ. As shown in Fig. S7a,b, the two band dispersions (*γ* and *ε*, indicated by the red arrows) forming the DP1 along the Γ(Γ’)-*K* direction are clearly observed in the second BZ, consistent with the experimental FS topologies in Fig. 2a.

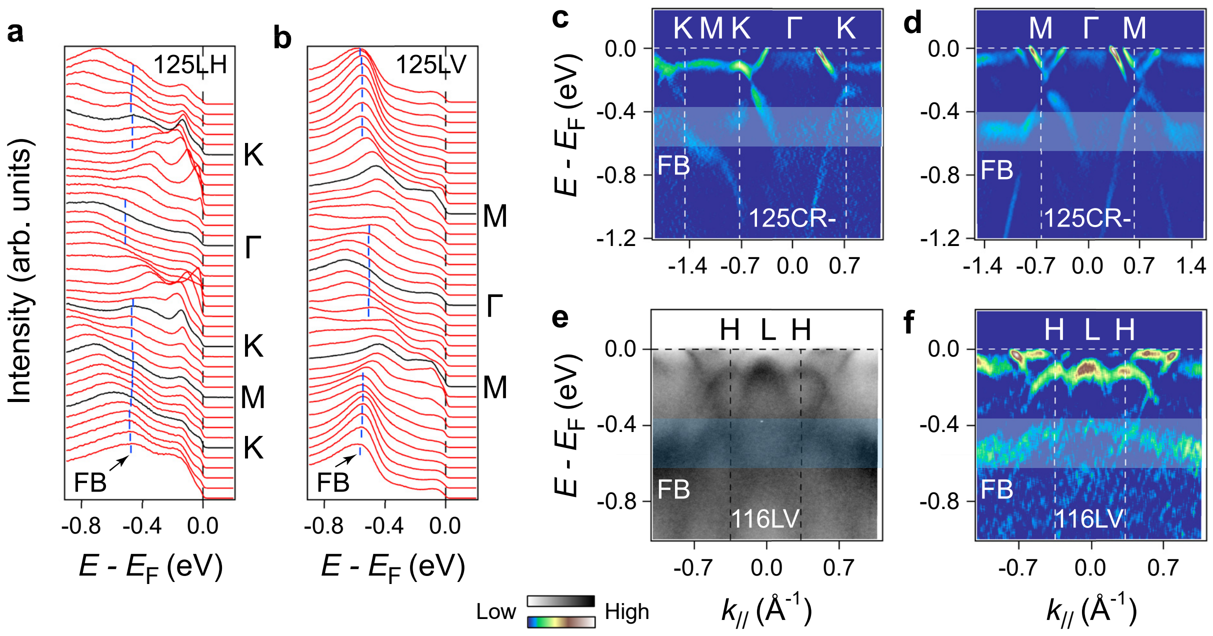

Figure S8 | Observation of the FB. a, EDC plot of the ARPES intensity taken along the Γ-*K*-*M* direction (*hv* = 125 eV, LH polarization). **b,** Same as **a** along the Γ-*M* direction (*hv* = 125 eV, LV polarization). The blue dashes indicate the FBs. **c,d,** Second derivative intensity plots taken along the Γ-*K*-*M* (**c**) and Γ-*M* (**d**) directions, respectively. The 125-eV photons with right-handed circular (CR^-) polarization were utilized. **e,f,** ARPES intensity plot (**e**) and corresponding second derivative intensity plot (**f**) along the *H*-*L*-*H* direction (*hv* = 116 eV, LV polarization). The FB regions are marked out by the shades in **c**-**f**.

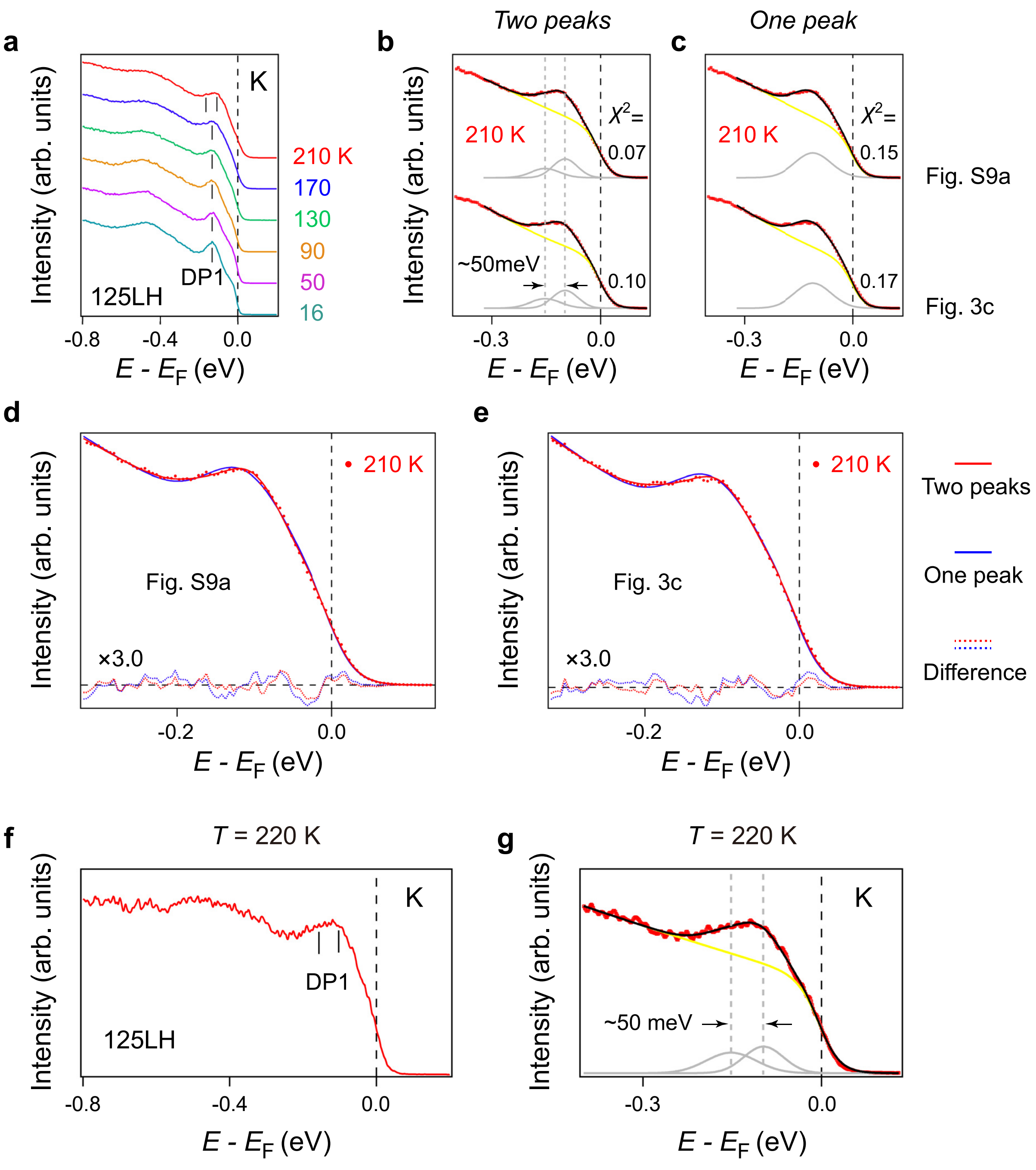

Figure S9 | Spin-orbit-coupling-induced (SOC-induced) gap at the DP1. a, Temperature-dependent EDCs taken at another *K* point of the first BZ. The black dashes are extracted peak positions. **b,** Quantitative fitting of the EDCs (210 K, red dots) at two different *K* points by using two Gaussian peaks (grey solid curves). The background (yellow solid curves) is modeled by considering a polynomial function together with the Fermi-Dirac distribution. The black solid curves are the fitting results. **c,** Same as **b** while the fits of EDCs are carried out by using one Gaussian peak. The chi-square (χ^2) value of each fit in **b** and **c** is also indicated. **d,e,** EDCs at 210 K from **a** (**d**) and Fig. 3c (**e**), respectively, together with the corresponding two-peak and one-peak fits. The dashed curves

represent the difference between raw data and fitting results. **f,** EDC taken at *K* point with the corresponding ARPES spectra recorded along the Γ-*K*-*M* direction ($h\nu$ = 125 eV, LH polarization, T = 220 K). The black dashes are extracted peak positions. **g,** Quantitative fitting of the data from **f** in the same fashion as **b**. The fitting result is superimposed as the black solid curve.

Supplementary Note 4: Reproducibility, temperature evolution, and quantitative analysis of the DP1 gap

To reproduce the temperature evolution of the DP1 gap, we performed similar temperature-dependent measurements at another *K* point of the first BZ. As shown in Fig. S9a, the temperature dependence of the line shape around DP1 is similar to that in Fig. 3c, demonstrating the reproducibility. In Fig. S9b, we plot together the EDCs measured at 210 K through two different *K* points. To quantitatively extract the gap size at the DP1, we fit the data close to E_F by using two Gaussian peaks and a background, which is modeled by considering a polynomial function together with the Fermi-Dirac distribution. The fitting results are superimposed as the black solid curves. An energy gap of ~50 meV can be obtained.

To validate the usage of two peaks in the quantitative analysis of the DP1 gap, we carried out the fit of EDCs (210 K) by using one Gaussian peak, as presented in Fig. S9c. In Fig. S9d,e, we plot the raw data together with the two-peak and one-peak fitting results. One can see obvious deviations between one-peak fit and raw data in the energy range from ~-0.1 to ~-0.2 eV, in sharp contrast to the overall goodness of the two-peak fit. This is clearly reflected in the difference curves (bottom dashed curves), where the absolute values of differentiation for the one-peak fit are generally larger than the two-peak one. To quantify the goodness of these two fits, we calculated the chi-square (χ^2) values. As shown in Fig. S9b,c, the χ^2 values of the two-peak fit are much smaller than that of the one-peak fit, further validating our assessments.

To support the trend of the DP1 gap evolution, we carried out similar ARPES measurements along the Γ-*K*-*M* direction at T = 220 K. As shown in Fig. S9f, the line shape of the EDC taken at *K* point is similar to that of 210 K (Figs. 3c and S9a), in particular the double-hump structure at DP1. In Fig. S9g, we fit the data near E_F in the same fashion as Fig. S9b. The obtained DP1 gap size (~50 meV) is comparable to the gap value at 210 K, confirming its trend with the spin reorientation.

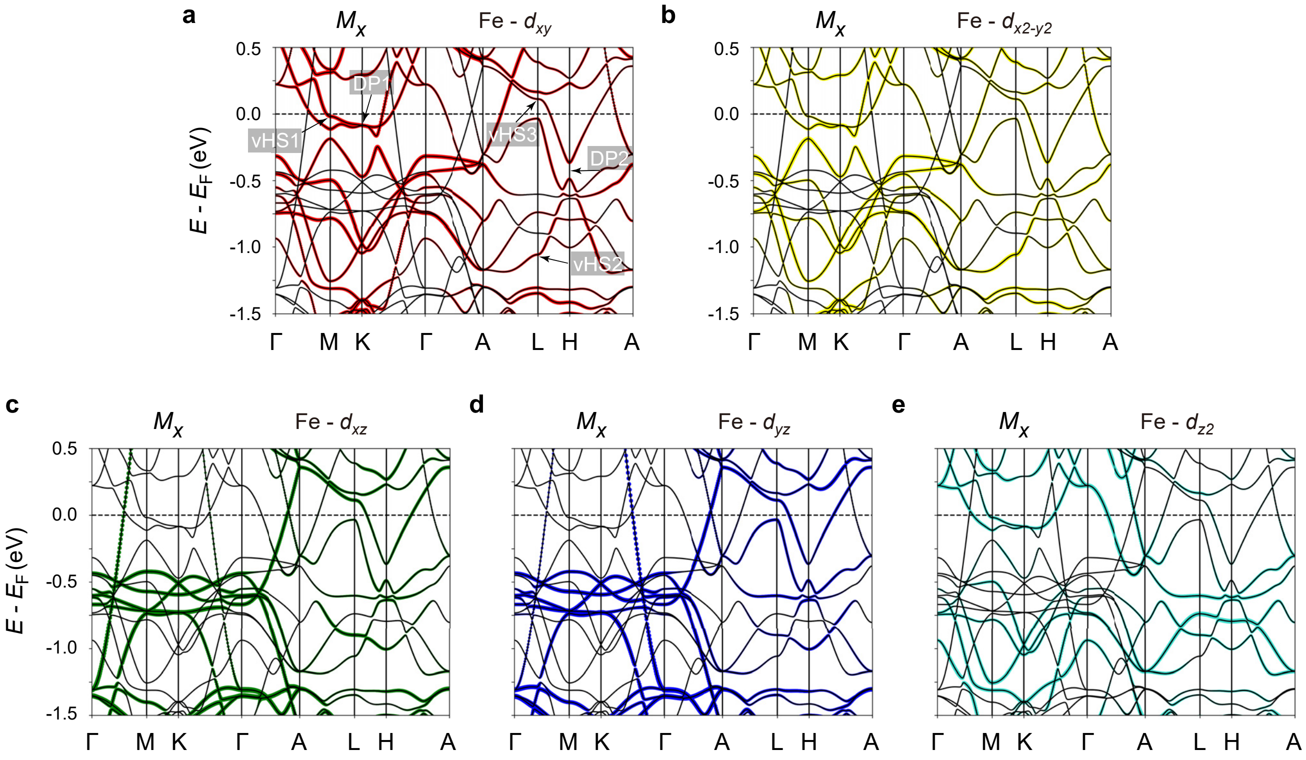

Figure S10 | Orbital-resolved Fe-3*d* band structure. a-e, DFT electronic structures of FM Fe_3Ge for the Fe moments ($\mu_{Fe} \approx 2.2\mu_B$) aligned along the *x* axis with spectral weight projected onto five Fe 3*d* orbitals, respectively. The red, yellow, green, blue, and turquoise colours represent the d_{xy} (**a**), d_{x2-y2} (**b**), d_{xz} (**c**), d_{yz} (**d**), and d_{z2} (**e**) orbitals, respectively. The DP1,2 and vHS1,2 below E_F as well as the vHS3 slightly above E_F are marked out in **a**.

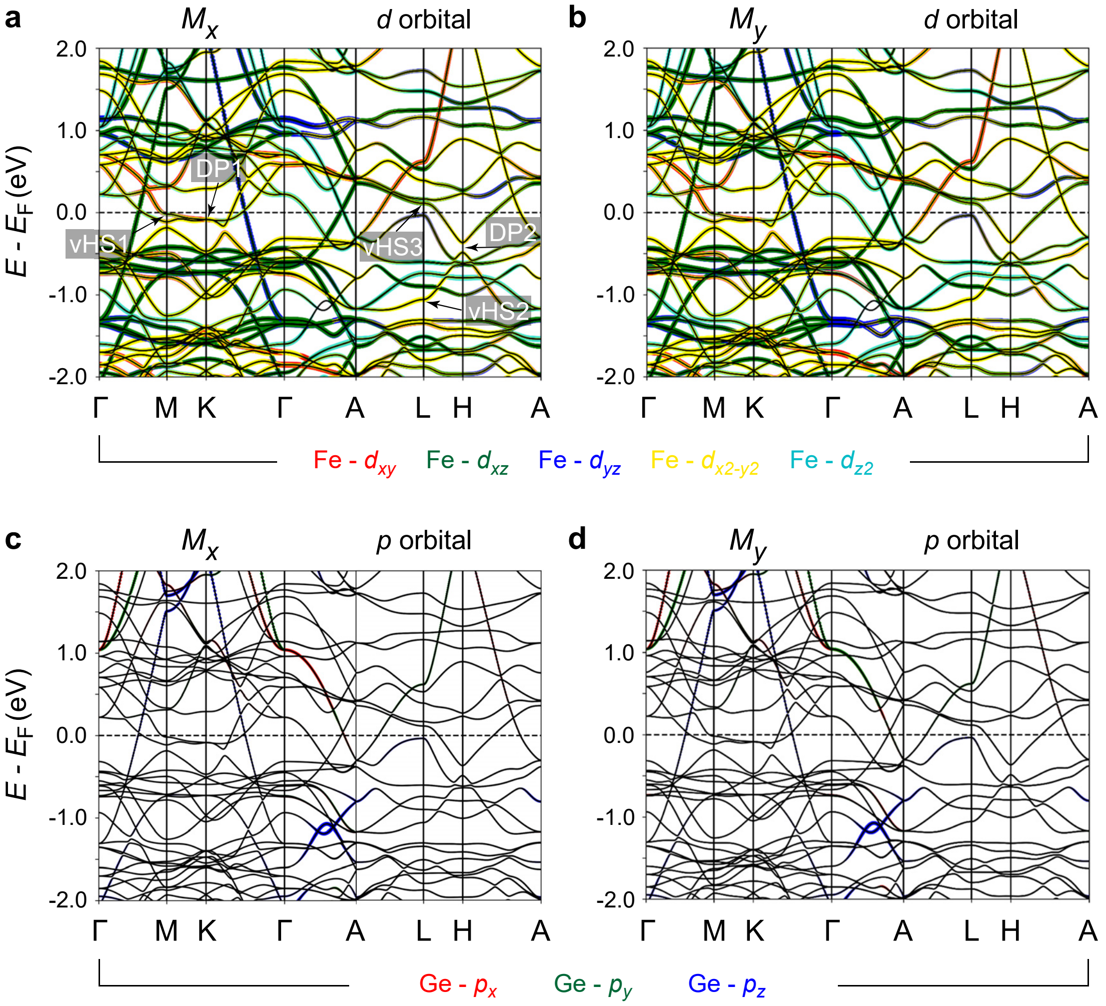

Figure S11 | Orbital-projected DFT calculations. a,b, DFT band structure calculations of FM Fe_3Ge for the Fe moments ($\mu_{Fe} \approx 2.2\mu_B$) aligned along the *x* (**a**) and *y* (**b**) axes with the Fe-3*d* orbital projections, respectively. The red, green, blue, yellow, and turquoise colours represent the d_{xy}, d_{xz}, d_{yz}, $d_{x2\text{-}y2}$, and d_{z2} orbitals, respectively. **c,d,** Same as **a,b** with the Ge-4*p* orbital projections. The red, green, and blue colours represent the p_x, p_y, and p_z orbitals, respectively.

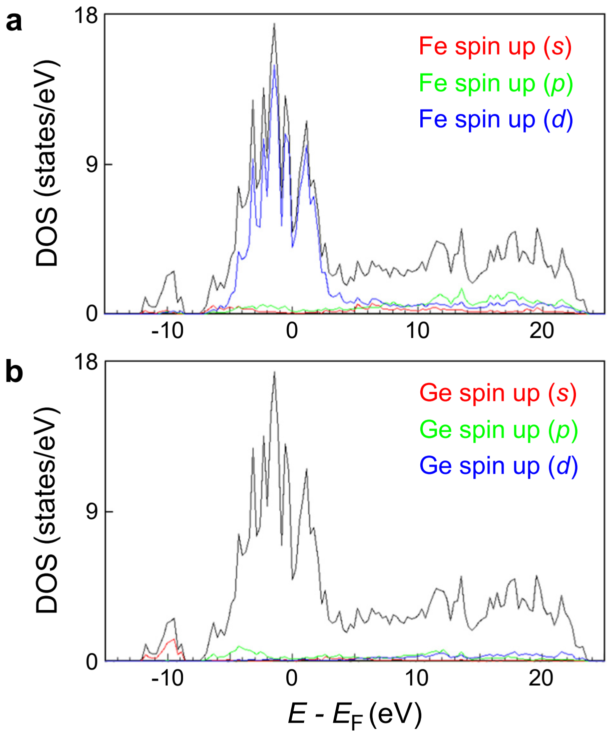

Figure S12 | Density-of-states (DOS) calculations. a, Calculated total (black curve) and orbital-resolved DOS of the Fe 4*s* (red curve), 3*p* (green curve), and 3*d* (blue curve) for the FM Fe_3Ge. **b,** Same as **a** but with orbital-resolved DOS of the Ge 4*s* (red curve), 4*p* (green curve), and 3*d* (blue curve).

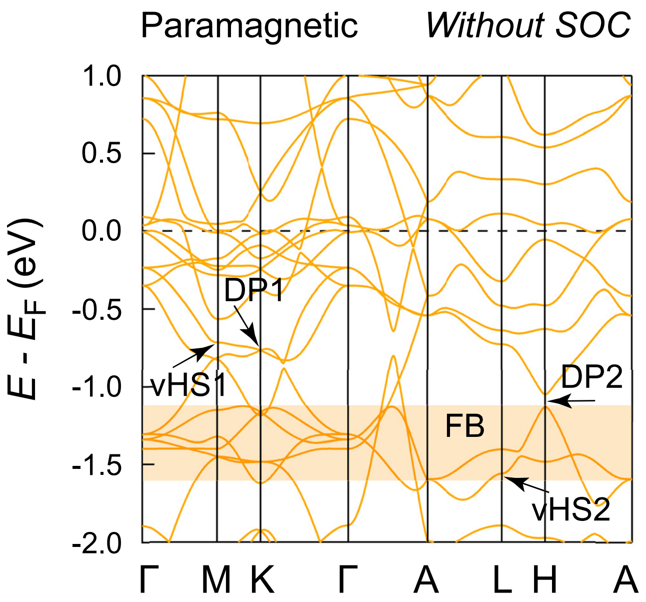

Figure S13 | DFT calculations without the SOC effect. DFT calculated band structure for the PM phase of Fe_3Ge without including the SOC effect. The DP1,2, vHS1,2, and FB region are marked out.

Supplementary Note 5: Negligible contribution of SOC to the Dirac gap at DP2

To understand the origin of the Dirac gap at DP2, we calculated the bulk band structure of PM Fe_3Ge without considering the SOC effect. As shown in Fig. S13, the DP2 gap at *H* point is still present, implying that it is not derived from the SOC. This is in sharp contrast to the case at DP1, where a gapless feature is observed without SOC (Fig. S13) and a gap opening is observed with SOC (Fig. S3a), respectively. Such evolution can further confirm that the Dirac gap at DP1 is of SOC origin. Consequently, we suggest that the SOC effects related to the $3d_{xy}/3d_{x2-y2}$ and $3d_{xz}/3d_{yz}$ orbitals, which form the DP2, should be negligible and contribute little to the predicted Dirac gap at DP2. Meanwhile, the negligible SOC effect of the $3d_{xy}/3d_{x2-y2}$ electrons also illustrates the dominant role of the $3d_{z2}$ orbitals in determining the SOC gap at DP1.

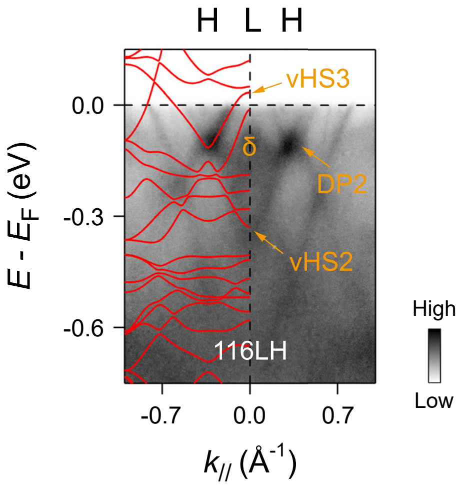

Figure S14 | Correspondence between the ARPES spectra and the renormalized DFT calculations. ARPES intensity plot measured along the *H*-*L*-*H* direction with the photon energy of 116 eV (LH polarization). The figure is adopted from Fig. 2d. The red curves are DFT calculated band dispersions renormalized by a factor of about 3. The DP2, vHS2,3, and δ band are marked out.

	v_F (eV·Å)				E_Δ (eV)	
Band features	*α*	*β*	*η* (*H*−*L* line)	*η* (*A*−*H* line)	|DP1 - vHS1|	|DP2 - vHS2|
DFT	2.03	1.25	1.63	1.30	0.068	0.69
ARPES	0.80	0.75	0.81	0.67	0.020	0.25
DFT/ARPES	2.5	1.7	2.0	1.9	3.4	2.8

Table S4 | Quantitative comparison between ARPES and DFT. The Fermi velocity (v_F) of the *α*, *β*, and *η* (along the *H*-*L* and *A*-*H* lines) bands and the relative energy shift (E_Δ) between the DPs and vHSs estimated from DFT calculations and ARPES experiments. One can obtain an overall renormalization factor of 2−3. It is noted that, for the better comparison with the experimental Fermi velocity, the E_F in the calculations was shifted to enable the energy positions of the *α*, *β* band bottoms and the DP2 to match the values in the experiments.

Supplementary Note 6: Examining whether the vHSs can induce electronic instabilities

In order to examine whether the near-E_F vHS1 (k_z = 0) and vHS3 (k_z = π) can induce any electronic instabilities in Fe_3Ge, we calculated the zero-frequency joint DOS by the autocorrelation of the experimental FSs in the k_z ~ 0 and k_z ~ π planes:

$$C(\boldsymbol{q}, E_{\mathrm{F}}) = \int A(\boldsymbol{k}, E_{\mathrm{F}}) A(\boldsymbol{k}+\boldsymbol{q}, E_{\mathrm{F}}) d\boldsymbol{k}\,, \qquad (1)$$

where $A(\boldsymbol{k}, E_F)$ is the spectral function at E_F at the $\boldsymbol{k}$ point in the BZ. The zero-frequency joint DOS describes the phase space for scattering of electrons from the state at $\boldsymbol{k}$ to the state at $\boldsymbol{k}$ + $\boldsymbol{q}$ by certain modes with wave vector $\boldsymbol{q}$. Therefore, one would expect that $C(\boldsymbol{q}, E_F)$ peaks at the corresponding ordering wave vector if there exists an electronic instability induced by the FS nesting. This autocorrelation of the ARPES spectra has been demonstrated to give a reasonable count for the charge-ordering instabilities of various compounds [2−6].

The autocorrelation maps in the k_z ~ 0 and k_z ~ π planes are presented in Fig. 5a,b. One observes that no peaks appear around *M* points in Fig. 5a, suggesting that the nesting between the vHS1 at *M* points is negligible, and thus is not able to induce the electronic instabilities. In contrast, as shown in Fig. 5b, the peak appears at *L* point where the vHS3 locates, indicating the existence of nesting between the vHS3 around the k_z ~ π plane. The anisotropic amplitudes between the nominally equivalent *L* points might arise from the matrix element effects in the ARPES spectra. These autocorrelation results further validate that the vHSs in kagome metals have orbital-dependent contributions to the electronic nesting, where the $3d_{xz}/3d_{yz}$ orbitals could be dominant. Although there could exist the nesting between the vHS3 in the k_z ~ π plane, as discussed in the main text, the 3D nature of Fe_3Ge could give rise to vHS3 being near E_F only in a small range of k_z. As a result, the nesting contributed by the vHS3 is most likely not sufficient to cause the charge fluctuations on the entire 3D FS. Therefore, the vHS3 could not induce the electronic instabilities either.

References

[1]. Göthelid, M. et al. Adatom and rest-atom contributions in Ge(111)*c*(2×8) and Ge(111)-Sn(7×7) core-level spectra. *Phys. Rev. B* **48**, 2012(R) (1993).

[2]. Shen, D. W. et al. Novel mechanism of a charge density wave in a transition metal dichalcogenide. *Phys. Rev. Lett.* **99**, 216404 (2007).

[3]. Chatterjee, U. et al. Nondispersive Fermi arcs and the absence of charge ordering in the pseudogap phase of $Bi_2Sr_2CaCu_2O_{8+\delta}$. *Phys. Rev. Lett.* **96**, 107006 (2006).

[4]. McElroy, K. et al. Elastic scattering susceptibility of the high temperature superconductor $Bi_2Sr_2CaCu_2O_{8+\delta}$: a comparison between real and momentum space photoemission spectroscopies. *Phys. Rev. Lett.* **96**, 067005 (2006).

[5]. Shen, D. W. et al. Primary role of the barely occupied states in the charge density wave formation of $NbSe_2$. *Phys. Rev. Lett.* **101**, 226406 (2008).

[6]. Cho, S. et al. Emergence of new van Hove singularities in the charge density wave state of a topological kagome metal RbV_3Sb_5. *Phys. Rev. Lett.* **127**, 236401 (2021).
